# Essays on the Effects of Iodine in Scrofulous Diseases, &c.

**Published:** 1832-01-01

**Authors:** 


**Essays on the Effects of Iodine in Scrofulous Diseases; including an Inquiry into the Mode of preparing Ioduretted Baths. Translated from the French of *M. Lugol*, Physician to the Hôpital St. Louis, by *W. B. O'Shaughnessy*, M.D. With an Appendix by the Translator, containing a Summary of Cases, &c. Octavo, pp. 218. Highley, October, 1831.**

Scrofula is a disease, or rather congeries of diseases, the importance of which, especially in England, will not be doubted; while the remedy treated of in the volume before us is comparatively new, and, we are strongly convinced, one of immense power in various maladies. The greater part of the work here presented in an English dress consists of three essays, published at short intervals (in 1829, 30, and 31) by Dr. Lugol, one of the physicians of the St. Louis Hospital in Paris. These essays were warmly eulogized by Magendie, Serres, and Dumeril, in their reports to the Royal Academy of Sciences, as well as by the medical press of France, while a prize of six thousand francs was voted to the author by the Institute; so that they come before the British faculty with no trifling testimonies in their favour. It is also to be borne in mind, that Dr. Lugol had every facility that could be desired for the benefit of his investigations—extensive wards dedicated exclusively to the treatment of scrofulous disease—and no limit to the period of residence. The results appear astonishing, as regards the success of the remedy; and the translator candidly confesses his belief that the amelioration may, in a few instances, have depended on other agencies than those of iodine; as the efforts of nature or the revolution of the seasons. Some of the cases, too, he thinks, though apparently cured, will ultimately relapse.

> "But admitting all this (and the concession does not extend to more than a small fraction of the cases), where is the competent practititioner, who, after a perusal of the following pages, will refuse to admit, that M. Lugol's treatment has in numerous examples rescued the patient from the alternative of the knife or the coffin, and in the majority has at least effected decided, though perhaps temporary, improvement."—*Preface*, viii.

The work is divided into three parts. The *first* is on the treatment of scrofulous diseases by iodine—the *second*, an inquiry into the effects and mode of employment of ioduretted baths in scrofulous diseases—and the *third* is a reiteration, or rather continuation, of the first—the treatment by iodine. The Appendix is alluded to in the title-page.

The report of Messrs. Serres, Magendie, and Dumeril to the Royal Academy, presents a very good coup d'œil of the work, and with it we shall commence our analysis.

"In the first place, we would remind our hearers that the scrofulous affections, long known under the names of 'cold humours,' or 'the evil,' constitute a class of those slow, unsightly, and often hereditary diseases, which strike despair into whole families, from the absolute rarity of their cure, and from the irremediable light in which they are regarded by the majority of physicians, and by the hospital regulations. Hence, also, the afflicted patients submit themselves to the illusive practices suggested by superstition; for, though medicine has successively tried all the remedies with which she is acquainted (the number, and even the absurd variety, of which attest too strongly the want of a certain method of cure), it must still be confessed, that up to the present time an efficacious mode of treatment remained to be made known.

Sometimes this disease is external and visible, and shows itself under the skin by swellings, which are slowly developed, become softened, burst, and remain ulcerated for a lengthened period, and thus produce callous and incurable scars; it takes its place in the substance of the integuments, which it renders deformed and disgusting; attacks the ears, the eyelids, the nostrils, and the lips, which become horribly tumid, or are corroded to such an extent as utterly to disfigure human nature.

Sometimes, more deeply hidden, the scrofulous habit attacks the bones and their articulations, obstructs the canals which transport the lymph and chyle, or produces in the lungs, and most important organs, tubercles which ultimately soften, and degenerate into purulent centres, thus giving rise to serious morbid alterations in the living economy, which eventually yields to the effects of the disease.

Such is an abridged view of the frightful malady to which M. Lugol, with zeal, perseverance, and success, has opposed a remedy, not absolutely new, but which had never previously been administered with so much method and precaution, to such a number of individuals at once, or with such evident and decided success.

M. Lugol is one of the distinguished physicians attached to the Hôpital Saint Louis, the only hospital in Paris where a great number of scrofulous patients are admitted for internal treatment. This circumstance explains how, in the short period of seventeen months, from the 10th of August, 1827, to the 31st December, 1828, M. Lugol has been enabled to collect the detailed cases of upwards of 100 patients; in whom he, of course, found great variety in the seat and intensity of the disorder.

Before your commissioners proceed to give an analysis of the memoir, they deem it right to declare, that they have not at all confined themselves to the scrutiny of its contents; but that they have seen, examined, and questioned the patients under treatment, and have also visited some of those reported cured or convalescent,—that all the author's assertions have been found scrupulously exact,—that many of the patients who were under treatment when the Memoir was finished, have since been completely cured.

Without restricting ourselves to the order followed by M. Lugol in his Treatise, we proceed to make known its principal results.

In the first place we may observe, that he uses two preparations of iodine: the one, exclusively intended for internal administration, is a solution of this simple substance in distilled water. The others are proper for external application, whether as ointments for ulcers, pomade for frictions, or watery solutions of varied strength, for collyria, lotions, and injections.

The motives which have induced M. Lugol to employ by preference the *aqueous* solution of iodine, appear exceedingly plausible. So active a medicine can scarcely be administered in an hospital without inconvenience and uncertainty, except in the form of a drink. The alcoholic tincture and syrup of iodine present many disadvantages in the exact measurement and distribution of their doses, while a pint, or half a pint, of distilled water, containing in solution a little common salt, and a fixed quantity of iodine, affords us an easy, precise, and economical method of dispensing the remedy. Two degrees of this solution intended for the patients, and designated by the name of 'Mineral Water' No. 1 and No. 2, the first containing two-thirds of a grain, and the second one grain of iodine in solution, have furnished the means of dosing exactly from day to day, and of recognising the effects of what was previously employed. Thus, half of No. 2 is the first allowance, the entire of No. 1 the second, and, finally, the whole of No. 2.*

As to the preparations intended for the external treatment, these are unctuous substances of a certain weight, and associated in determined and successively increasing proportions with iodine, ioduret of potassium (hydriodate of potash), or with the proto-ioduret of mercury.

These simple means have sufficed M. Lugol for the treatment and cure of numerous cases, twelve of which, selected from the different species of scrofulous affections, are described in the Memoir. Three relate to ulcerated tubercles, cured in three, seven, and twelve months. Two cases are also described of ophthalmia and coryza, one of which yielded to a treatment of forty-six days, while the other was prolonged to the ninth month. A case of fistulous abscess deeply situated in the cellular tissue, has required nearly a year's care. Four cases are also recorded by M. Lugol, of that frightful form of the disease most usually denominated 'dartre rongeante,' but which the author names the *esthiomenic* (or corrosive) scrofula. Finally, a case of scrofulous caries is detailed. This last form has generally been found very intractable. M. Lugol is only able to advance this single case of cure. It will be remarked, also, that the proto-ioduret of mercury was used, and that there still remains a small fistula as yet unhealed, but which appears to have a tendency to cicatrisation.†

All these cases are given at great length, they present complete accounts of the history and symptoms of the patients at the time of their first examination, and before the treatment was commenced. Many of the cases have been figured, and a record is presented of the modifications which have supervened during the treatment, as noticed twice every month, until the cure or discharge of the individual.

The author of the Memoir has carefully noticed the effects produced by the iodine on the animal economy. Applied externally, its local action has always been very sensible : it determines on the surfaces of the ulcers a feeling of smarting, accompanied with painful itchings. *This application to the diseased surfaces changes their appearance, and frequently produces as appreciable an effect as that determined by mercury on venereal ulcers.* Moreover, the mode of its action does not appear to be invariably the same : sometimes the iodine seems to melt down and resolve the tubercles, sometimes, on the contrary, it urges them

---

* "It will be seen, in the third part of this Memoir, that the author subsequently altered his formula, and substituted for it a solution of iodine in the hydriodate of potash. The first prescription, however, cannot be omitted here, so closely is it interwoven with the treatment of the twelve interesting cases in the first Memoir. Additional reasons, stated in the Appendix, concur to render the account of this simple solution of iodine a subject of considerable importance.—Translator's Note."

† "In the Third Part of the Treatise many additional cases are recorded of the successful treatment of caries, hypertrophy, and spontaneous luxation of the bones.—Translator's Note."

on to rapid suppuration. At other times the painful sensation appears to diminish in proportion to the healing of the surfaces, an effect which is perhaps dependent on habit; nevertheless, some ulcers remain sensible while the curative process is not at all established.

Internally administered, and always in small doses, and with the most prudent slowness, the ioduretted water constantly excites the appetite, and appears to increase the urinary and salivary secretions. Sometimes, but very rarely, it has become purgative to so considerable an extent that its use was necessarily suspended, at different intervals, from two to three days each. In other and still rarer cases, in which the solution of iodine appeared to occasion pain in the stomach, the wine of quinquina, given according to the directions of M. Coindet, in a dose of two or three ounces, put an end to the troublesome symptoms. M. Lugol, however, always declined as much as possible this association of remedies, in order to avoid complexity in the results of his treatment.

Iodine, administered in this diluted form, has never caused emaciation nor produced the expectoration of blood or other accidents, which many have imputed to its action.

From the contents of the first Memoir it appears that M. Lugol has treated with iodine alone, in seventeen months, at the Hôpital St. Louis, 109 scrofulous patients, of which 61 were males and 48 females.

That at the close of last year, 39 (29 males, 10 females) were still under treatment.

That 30 (17 males, 13 females) had quitted the hospital with marked improvement.

That in four cases (2 males, 2 females) the treatment was ineffectual.

Finally, that 36 (13 males, 23 females) were discharged completely cured.

The author concludes, from all the facts he has collected, and the researches he has conducted, that iodine deserves to be considered as the most efficacious remedy in scrofulous diseases, since it has constantly arrested their progress, or at least exercised a salutary action in the treatment of all tubercular tumours, even when it has not evidently accomplished their cure. He therefore believes that the introduction of this remedy into medicine is one of the most valuable acquisitions the healing art has made in modern times.

We shall then confine ourselves to say, that after having made ourselves acquainted with the facts cited in the memoir, we have been enabled to confirm the evident action of the remedy; and that we believe M. Lugol to have effected a work of great utility by availing himself of the facilities afforded by his situation, in seeking for a remedy for a disease hitherto so deplorable and desperate. We consequently propose to the Academy to encourage this physician to persevere in the researches which he has hitherto pursued with so much zeal and sagacity.

(Signed) SERRES,
MAGENDIE, and
DUMERIL, Reporter." 9.

The first chapter is dedicated to an enquiry into the pharmaceutical preparations of iodine. After some remarks on Coindet's tincture of iodine, and M. Henry's syrup, the author states his preference to the perfect solution in distilled water.

"At first I fixed this vehicle at a pound, in this I dissolved half a grain, two-thirds of a grain, or a grain of iodine, in order to have at my disposition three degrees of the same remedy, to be used according to the individuals and periods of treatment."

"I have denominated these three degrees of the solution as ioduretted mineral water, No. 1, 2, and 3. In all cases I commenced by No. 1, seldom proceeding to 2 till the second month of treatment. I have by no means given No. 3 to all scrofulous patients, and I have never had occasion to pass this last dose of one grain per day." 12.

By this it appears that M. Lugol never ventured so far as M. Coindet in the doses of iodine, who gives it to the extent of three grains per diem. He objects to the tincture of iodine, because, when put into water, the iodine is precipitated in the pure solid state, and thus may produce intense excitement in the stomach.

"But whatever merit may be possessed by the preparations thus recommended for internal use, they do not answer a purpose of frequent occurrence in scrofulous cases, viz.—that of local treatment. I, therefore, at first, prescribed a particular ointment, of three different strengths, composed of hydriodate of potash and iodine.

IODURETTED OINTMENT.

| | No. 1. | | No. 2. | | No. 3. |
|---|---|---|---|---|---|
| ℞. Fresh Lard ........ | ℔ij. | .. | ℔ij. | .. | ℔ij. |
| Hydriodate of Potash | ℥iv. | .. | ℥v. | .. | ℥v. |
| Iodine ............ | ʒiv. | .. | ℈xiv. | .. | ℈xvj. |

Afterwards I made use of a solution of iodine, which occasionally forms a valuable substitute for the preceding ointment, especially in scrofulous ophthalmiæ, and for the injection of fistulous canals.

| | No. 1. | | No. 2. | | No. 3. |
|---|---|---|---|---|---|
| ℞. Iodine .............. | gr. ij. | .. | gr. iij. | .. | gr. iv. |
| Hydriodate of Potash | gr. iv. | .. | gr. vj. | .. | gr. viij. |
| Distilled water..... | ℔j. | .. | ℔j. | .. | ℔j." |

During the early part of his experience, M. L. dressed ulcers or rubbed tumours twice a day; but as this practice was often followed by irritation, he now limits the operation to once in the 24 hours, except in particular cases of profuse suppuration.

### I. External Local Effects.

"The external employment of iodine ordinarily produces intense local action, and often causes a prolonged sensation of prickling and smarting, especially severe on bathing days.

In many cases this action terminates by a fit of itchiness, short in proportion to the duration and the degree of pain first experienced.

A few days are sufficient to change the aspect and improve the condition of ulcers, whether suppuration be produced or not." 15.

Illustrative examples are given from practice. The author avers that it is no exaggeration to say that "iodine changes the appearance of scrofulous ulcers sometimes more quickly than mercury modifies that of syphilitic sores." He has seen ulcers too quickly cicatrized—that is before the complete resolution of the tubercles. The skin, when rubbed with iodine, becomes of a reddish yellow colour, from absorption, its presence in the cutaneous tissue, and its injection into the capillary vessels. The epidermis soon becomes detached in layers, so that the ointment comes into immediate contact with the true skin.

### II. Internal Effects of Iodine.

"The internal use of iodine frequently produces particular effects; one of the most important noticed at St. Louis was the increase of appetite in the patients to such an extent that the hospital allowance of food was scarcely or not at all sufficient. This is certainly one of the best effects of iodine, for not only does it indicate an improved state of the digestive organs, but it enables us with ease to invigorate the general constitution by wholesome nourishment, which is particularly valuable in scrofulous patients, in whom very frequently the appetite is almost entirely deficient.

This ordinary effect of the ioduretted preparations on the animal economy sufficiently denotes the numerous applications which may be made of them in the treatment of other diseases, here unnecessary to enumerate, but in which the digestive organs require to be excited.

Iodine is a powerful diuretic.* All the patients using it have informed me that they pass urine copiously; and I have known this secretion to be so much increased with many that they were obliged to rise once, twice, thrice, or oftener, by night, than was their usual custom; some have even experienced this diuretic action of the ioduretted mineral water in so instantaneous a manner that iodine was detected in their urine almost immediately after the dose was taken.

More than one-third of the patients who used it have experienced a purgative effect also, and in this respect there prevailed much diversity, from mere freedom of the alvine evacuation, to six or seven stools daily."

"This purgative action of iodine, when kept up to a certain degree, prevented my increasing the dose without much caution; but it never caused me to suspend the internal employment of the remedy, except for intervals of two or three days, as I did with the ointment, when it smarted too powerfully.

Iodine has also produced, in several instances, remarkable salivation. I have seldom observed that effect but in *male* patients. It was especially remarkable in Poiré (see Case X.), who was salivated most profusely in the morning after drinking the mineral water. The ioduretted frictions also operated remarkably in this case.

Several patients, the females especially, have complained of pain in the stomach. I have always stopped this uneasy symptom with the kina wine, of which the patients took two or three ounces after their mineral water. On this point, M. Coindet's experience anticipated mine; and my observations, on the other hand, have verified those of that excellent practitioner, on the efficacy of kina in appeasing the cardialgic affections sometimes produced in certain individuals by the use of iodine." 20.

Although it is desirable to test the effects of iodine by administering it separately; yet there is no reason why we should not combine it with various other remedies, in particular cases, and to answer various indications.

The author denies that iodine injures the health in any way, or even causes emaciation, when properly administered. He has never seen an instance of emaciation from its use.

"Far from being ever hurtful, it is a powerful stimulant which revives the organic functions, fortifies the general constitution, and *encourages the growth* and *increase of size.* I have drawn up a statistical account of the scrofulous females treated in the course of eighteen months, and I may cite here as the general results: 1. *That thin females* have acquired a state of *embonpoint.* 2. That cor-

---

* We believe we were among the first to notice this effect of iodine, in this country. Mr. Guthrie and the Editor of this Journal witnessed the extraordinary diuretic effects of iodine in the case of a gentleman, who perfectly recovered from ascites and diseased liver, after being twice tapped, and being on the eve of death from visceral disease and serous effusion in the abdomen.—*Ed.*

pulent women have not become emaciated. 3. That those not belonging to either of the preceding heads, have lost nothing of their middle state, but have gained increased strength and improved health." 23.

In the succeeding section Dr. Lugol maintains that iodine does not produce pulmonary tubercles, hæmoptysis, or other accidents apprehended by some practitioners. On this subject we need not dwell, as few practitioners in this country entertain any such apprehensions.

The second chapter is dedicated to a narrative of cases illustrating the efficacy of iodine in tubercular scrofula—in ophthalmia and scrofulous coryza—in scrofula of the cellular tissue, or scrofulous abscess—in cutaneous scrofula—in corrosive scrofula—and in scrofulous caries. On these we cannot enter; but strongly recommend a careful perusal of them by every practitioner. While the author sets forth the extraordinary success of the remedy in various diseases—a success which was witnessed and which excited the admiration of numerous foreigners in the Hôpital St. Louis—he does not silently slur over "the cases, too numerous, which have proved refractory to the remedy;" but properly animadverts on the evils inflicted on science by the abuse of "universal propositions."

### Part II.

This embraces an inquiry into the effects and mode of employing ioduretted baths in scrofulous diseases. We are informed by the author that metallic baths decompose the iodine, and therefore he uses wooden troughs, which exert but little action on the solution.

"My first formula was composed of one ounce of the hydriodate of potash, and an half ounce of iodine, dissolved in twenty ounces of distilled water, and next diluted in the quantity of water necessary for a bath; but this bath having produced intense rubefaction of the skin, I reduced it in the sequel one-fourth, and composed the bath for an adult with six drachms of the hydriodate of potash and three of iodine.*

With this formula, which is the strongest I at present employ, I have administered the ioduretted baths to a great number of persons. The following observations are of importance to be attended to.

The lady first experimented on had still her skin too intensely reddened.

A young lady of 17 could not support it; her skin was reddened, especially at the neck; and what was evident to the view, and well worthy of remark, was, that the skin of the neck where it was tubercular was more reddened than at the opposite side.

A young lady of 18 (see Case V.) could not endure the bath at this strength.

Another, æt. 7, found it also too irritating, and on one occasion could not be kept in the bath.

The child of four years old (the subject of the third case) was powerfully rubefied, and had its penis inflamed by a bath composed of five buckets of water, and one-fourth of the iodine solution prepared for an adult.

---

* "The hydriodate of potash, as was first ascertained by M. Bauf, apothecary at Vevay, can, in concentrated solution, dissolve twice as much iodine as it contains itself (once and a half its own weight); but as this solubility of iodine diminishes in proportion to the increase in the quantity of water, I have adopted the proportions recommended by this apothecary, namely, one part and a half of iodine, to one of hydriodate of potash. It, besides, appeared to me useful not to change an established formula, and one generally followed.—Author's Note."

A school-boy, æt. 15, was powerfully rubefied, as well as his father, who stirred up the bath with his arm, in order to render the solution uniform, to estimate its temperature, and to judge, by his own sensations, if the bath smarted so severely as his son asserted.

M. ——, æt. 25, had the skin much reddened, and that of the penis inflamed for several days, so much so that he was feverish at one interval for thirty-six hours.

Now, the bath which produced these accidents contained about three drachms French, or about 216 grains of free iodine for 240 quarts of water, that is, about nine-tenths of a grain per quart. But I have long since been in the habit of administering internally, without inconvenience, a grain of iodine daily in twelve ounces of the vehicle, to the majority of my scrofulous patients in the second period of their treatment. I daily caused the eyes, nose, and lips, to be bathed, and fistulous channels to be injected with a solution containing three, four, or five grains of iodine to the pound of distilled water.

In certain scrofulous diseases of the skin, tubercles, cellular tissue, &c., I have found an ioduretted solution of half an ounce of hydriodate of potash and two drachms of iodine, in eight ounces of water,* to act as a powerful rubefacient and caustic.

Now, if we compare the proportion in which the iodine exists in an ioduretted bath, with the dose contained by the other preparations of which we have spoken, the energy of the baths will appear much more surprising. The quantity, compared with that of the mineral water for drinking, is only as one-third; compared with that for bathing the eyes, nose, and lips, it is little more than a seventh, ninth, or eleventh. Relatively with the solution which I mix with the cataplasms, or which I apply alone, to produce rubefaction in certain conditions of scrofula, it is only 1-640th. On these data, who would, *à priori*, have imagined that nine-tenths of a grain to the quart of water could have produced so decided an effect on the surface of the body? Nevertheless, such was the effect that I have not been able to resume the first formula, and that I have been obliged to substitute for it two others of lower strength; the first of these contains five sixths, the second four-sixths, of the original quantities. Except under particular circumstances, I commence with the former." 60.

The author proceeds to point out the difference between hydriodate of potash and iodine in ioduretted baths. The *former* he only considers as a solvent of the *latter*, and in nowise participating in the therapeutic agency of the ioduretted baths. He has employed the hydriodate alone, and gradu-

---

* "I have prescribed this ioduretted liquor for the past nine or ten months, to be added in sufficient quantity to poultices of linseed-meal, which I have applied to excessive growths, and scrofulous caries.

It serves equally for local baths to the hands, feet, chin, &c., by adding a certain quantity of it to the necessary proportion of water.

It is also very useful for touching certain surfaces which require excitement, especially palpebral ophthalmiæ, ozœna, ulcers, and the extensive surfaces in the corrosive or esthiomenic form of the disease.

In the cases where I found it necessary to touch more deeply I used a formula composed of six drachms of iodine and four of hydriodate of potash, dissolved in the smallest possible quantity of fluid.

I no longer employ any other mode of cauterisation for any case of scrofula; I have particularly applied it to the edges of the esthiomenic kind when it appears spreading, and also to a particular kind of pustules which remain insulated by the cure of other adjoining pustules, and which, unless their defective mode of vitality be altered, are exceedingly tedious in their cure.—AUTHOR'S NOTE."

ally increased its quantity to three ounces. He also employed the iodine, in the proportion of three drachms to each bath. Lastly, he tried the effects of the mixed solution of the two. He subjoins the names of seven patients on whom the experiments were made, together with a summary of their diseases. These cases and experiments we cannot analyse, and they are too numerous to insert in an extract. We shall, therefore, give the summary or conclusions to which the author came.

"1. The hydriodate of potash has scarcely any action whatever in the dose of three ounces to each bath.

2. Iodine should be regarded as the active principle of the baths.

3. The proportion of iodine should generally be from two to three drachms a bath, and very seldom beyond that.

4. Pure or simple iodine is not completely soluble in a bath; and in that case, its action being unequal, may give rise to local accidents; and it may also be deficient in its general action on the economy.

5. Iodine previously dissolved in alcohol does not continue in a state of solution when diluted with the bath, and it moreover produces olfactory phenomena, which may proceed to a species of drunkenness, or even to decided and durable cerebral congestion.

6. The most certain mode of preparation is the preliminary solution of the iodine in the hydriodate of potash.

The efficacy of ioduretted baths in scrofulous diseases is, I believe, already demonstrated by the consequences which naturally flow from the notions I have published on the use of iodine in these diseases. But besides their general utility, these baths still offer peculiar advantages—that of a substitute for the internal treatment, the doses in which may be diminished to different degrees according to the necessity of the case, and that of affording us a mighty auxiliary in certain cases of scrofula, which the internal and external modes of treatment hitherto known, have only altered in the most tedious manner." 76.

This part of the work is terminated by some narratives of cases, to which we beg to direct the attention of practitioners.

### Part III.

### On the Treatment of Scrofulous Diseases by Iodine.

This memoir is dated the 31st of May, 1831; and, after stating that he had continued his experiments and observations at the Hôpital St. Louis with increased success, he had thought it proper not to narrate cases on his own authority alone, but to solicit the Academy of Sciences to appoint a committee of examination. Messrs. Dumeril and Magendie having been accordingly appointed, they visited the hospital, and after mature deliberation and reiterated observation, reported as follows:—

"By the solicitation of M. Lugol, the Academy has appointed a commission to visit the Hôpital St. Louis, and there investigate the alleged advantages produced byiodine in the cure of the most serious scrofulous diseases. M. Dumeril and I having been entrusted with this honourable office, we proceed to lay before you the result of our inquiries.

The Academy is already acquainted, from our previous report on this subject, with the success of M. Lugol's practice; a success so remarkable and valuable, that a disease of the utmost prevalence, especially among the poor, and formerly of such protracted and difficult treatment, that it is excluded from our hospitals by still-existing regulations, has now become curable in a limited period, and by means of trifling expense; and thus the numerous paupers afflicted with it, now have a right to be admitted into the hospitals, and treated as ordinary cases.

The new facts which on the present occasion your committee has verified, are such as to remove every doubt on this subject. Not only have we witnessed the cure of scrofula in the first and second degree, but we have also seen the successful treatment of the disease in its most aggravated forms.

Deep-seated alterations of the glands and various other organs, serious lesions of the bones and their principal articulations, accompanied by those general symptoms which forebode a speedy death, have been perfectly cured, in great numbers of cases, in the space of a few months, leaving the patients in the best possible state, and free from every vestige of the malady except the ineffaceable scars it had originally effected. Moreover, these results are rendered still more valuable by the fact that the majority of cases subjected to M. Lugol's practice were, previously, in a desperate state, and only admitted into his wards as deplorable examples of the ravages of an irremediable disease. Among the unfortunate persons thus afflicted are frequently seen some whose mutilations are truly frightful. Before the discovery of iodine, they were all devoted to inevitable destruction, but since the introduction of that remedy and of bromine into therapeutics, one of your committee has had the happy satisfaction of restoring to life and comfortable existence many of those cases hitherto deemed of an incurable kind. It may not be superfluous to add that these cures have been as rapid as unexpected.

We shall not here enter upon an analysis of the individual facts submitted to our examination and authentication by M. Lugol. We have added some to this report, but they are not suited to be read to this assembly, for pictures so melancholy, without promoting the interests of science, could not fail to be disagreeable—one remark is nevertheless essential. In cases of tumours of the articulations, with caries or other alterations of the bony tissue, instead of recommending absolute rest, according to the general practice of surgery, M. Lugol includes regular exercise in his remedial measures. The cases of this kind which he has shown us leave no doubt of the advantage to be obtained in following this departure from the general rule.

We have already said, in our preceding report, that M. Lugol does not pretend to the discovery of the utility of iodine in scrofulous diseases ; but from the great number of cures he has obtained—from the zeal and perseverance with which he pursues his researches ; from the light he has thrown on the varied effects of the different preparations of iodine, internally and externally administered, it is manifest that he has contributed largely to the advance of medical science. And as, moreover, he has the wisdom to shun all idle and profitless speculations, the uselessness of which constitutes but their least inconvenience, we have the honour to propose that M. Lugol's researches receive your approbation, and that he be requested to continue to prosecute inquiries fraught with so much value to mankind.

DUMERIL.
MAGENDIE, Reporter." 88.

The author then proceeds to a detail of numerous cases, arranged under five distinct heads, viz. tubercular scrofula—scrofula of the mucous membranes, especially ophthalmia—cutaneous scrofula—scrofula of the cellular tissue—scrofula of the bones. To give any thing like an analysis of these cases would far exceed our limits; and yet we are loth to pass them entirely over in our Journal. We shall therefore select a case from each of the divisions above-mentioned, as specimens.

### I. Scrofulous Tubercles.

Case 1.—" *Ulcerated Tubercles of the Neck at each side—Deep Alterations of the Skin—Ophthalmia of the Right Eye—Copious Epiphora—Seven Months' Iodine Treatment—Cure.*

Philippe Jean Duport, shoemaker, aged 19, was admitted on the 6th May, 1829. At each side of the neck, from the upper extremity of the sterno-cleido-mastoid muscle to the back of the chin, there existed a great number of ulcerated tubercles, and the cutaneous tissue was also much diseased. The tubercles first appeared when he was 15 years old, in the Spring. Every year since, at the same season, additional tubercles appeared and the old ones increased in size. Ulceration supervened about three months after their first appearance. There was also a fluctuating tumour on the top of the sternum, and another, of hard consistence and about half the size of a nut, before the right masseter muscle. Both these were but of two months' duration. The ulcers all looked very badly, and were covered with thick grey crusts, which were occasionally separated by the discharge from the subjacent ulcers.

I may here remark, that scrofulous pus does not give rise to incrustations, so that those formed in this instance depended on the pus proceeding from the surrounding skin,* and they were detached almost as soon as formed, by the abundance of the tubercular discharge. The separation of the crusts in this case was accelerated by the slightest motion of the neck, and, by giving rise to trifling local hemorrhages, increased the hideous aspect of the patient on his first admission.

At seven years of age he had a fall, six months after which an immense abscess formed in the calf of the left leg, which would have been amputated at the Hôpital des Enfans, had not M. Jadelot fortunately opposed the operation. For this abscess he was six months confined to bed, and six months more during convalescence unable to walk. While the cellular, and perhaps the osseous, tissue was thus deeply affected, ophthalmia attacked both eyes, and continued for five or six months. At 18, during the active growth of the tubercles, ophthalmia of the left eye again occurred, which lasted four months and returned again two months before his admission. For many years he was subject to copious epiphora at both sides. The cause of the disease could not be traced to any hereditary predisposition; his parents were young and healthy, and his brothers free from any scrofulous affection. He followed his trade in Paris for a year in the Rue de Porton ou Marais, in a back room on the ground floor, where the sun's rays never entered. He also slept in this room. According to the custom of authors this would have been deemed a sufficient cause of the disease, but a more rigorous analysis of the case shows that the locality only acted as an occasional cause in its development of the predisposition to scrofula already inherent in this individual. The chronic abscess and the ophthalmia occurred while the patient was but a child, and lived with his parents in sufficiently comfortable circumstances, and not exposed to moist air. Besides, after the first eruption of the cervical tumours the patient left Paris and returned to his native place, where, though his circumstances and abode were favourable to his recovery, the disease nevertheless proceeded with scarcely diminished violence. Had moisture been the cause, when the patient was removed from its operation, the effects might reasonably have been expected to disappear.

1st August, 1829.—After twenty-six days' treatment with the ointment containing the proto-ioduret of mercury, and with the ioduretted mineral water, the tubercular ulcers were cicatrized, and tubercular matter was felt no longer in

* " In a state of *common* inflammation."—Translator.

any situation, excepting the two swellings which last appeared, and over which the cicatrices were less firm and healthy than in the other places. During the first month of the treatment the suppuration was very profuse; and for a period of nearly three months the local action of the iodine was most acute, causing severe pricking pains for two or three hours morning and evening.

16th October.—For more than two months the patient was only dressed once daily. No local suppuration or tubercular incrustations; the cicatrices of the two recent ulcers not yet sufficiently healthy. The iodine had by this time lost nearly all its local power.

During more than four months the ioduretted mineral water produced salivation; and from time to time it seemed to occasion one or two additional alvine evacuations daily. Slight diuretic action was also manifested. The appetite, ever good, was not impaired by the remedy. On the 28th February he was dismissed cured. He soon afterwards returned to the hospital in the capacity of a sick servant, and, during the events of July, applied himself with such zeal to the assistance of the wounded that he fell ill of fever and erysipelas of the face and scalp. Under the influence of the ordinary remedies he recovered in three weeks; and though his convalescence was protracted, no relapse of the scrofula took place, an event which was much apprehended.

In three months he obtained a more healthful and easy situation, and quitted the hospital in perfect health." 94.

### II. SCROFULOUS OPHTHALMIA.

CASE 2.—"*Purulent Scrofulous Ophthalmia in the most violent Form, and intense Coryza, mitigated in a few Days by active local Treatment with Iodine, and finally cured by the internal and local Method.*

Antoine Cretenet, æt. 16, of diminutive stature, entered the Hôpital St. Louis, on the 4th of May, 1830, for a double purulent ophthalmia and scrofulous coryza of the worst kind. He kept his head bent down and resting on the chest, and his eyes were covered with a triple bandage, in order to avoid the least contact of the light. On examination we found the eyes bathed in pus, and enlarged to an enormous size by the hypertrophy of their soft parts. The eyelids and their circumference were swollen, of an erysipelatous redness of the worst appearance; between their free edges there existed a large red, granulated chord formed by the conjunctiva, which had already acquired a thickness of two or three lines.

The swelling of the soft parts, and the exquisite pain occasioned by the contact of the light, although the eyelids were closed, did not allow us to attempt to open them in order to examine the cornea. We, however, regretted this the less, as we could only learn by the inspection the *degree* of violence of the disease, by which information the nature of the treatment could not be modified.

The nose participated also in the condition of the eyes, and was almost buried in the swelling of the surrounding soft parts. The nasal fossæ were choked with crusts, the alæ nasi were hypertrophied, so that the patient was obliged to breathe exclusively through his mouth. Both these affections were of about 13 months' duration, and their actual state had lasted for eight days. He had previously been affected with ophthalmia several times; had also laboured under obstinate chilblains, and had had favous pustules on the scalp. His father, a man of weakly constitution, had long suffered from ulcerated legs, and was some years dead. His mother died at 40, having ever been of a delicate and sickly habit. Of two brothers and two sisters, one of each died young, the others were in a state of wretched health, subject to chilblains, and constant swellings and incrustations of the nose and pituitary membrane.

The case appearing very urgent on admission, the acting-surgeon applied a blister to the neck, with mustard-baths to the feet, and a laxative injection. It is, however, notorious that this derivative method has failed to cure purulent ophthalmia in the practice of the most renowned physicians. It being also well known that this ophthalmia not unfrequently causes the destruction of the eyes in a few days, and from my conviction that the disease was here of a scrofulous nature, I did not hesitate to have immediate recourse to energetic local treatment with iodine. Whenever scrofula exists, iodine must be called in to our assistance.

5th May.—Local baths, ioduretted solution to be injected beneath the eyelids and into the nostrils with a small syringe. As the danger of losing the eyes was imminent, I stationed another patient near Creteret, to keep the local baths and injections constantly renewed.

7th May.—Pain and suppuration diminished, and the soft parts less impregnated with pus. By slightly opening the eyelids we found the cornea red and swollen.

10th May.—Swelling of the soft parts much decreased, the patient can open his eyelids and support the daylight. The secretion less abundant and not so yellow. He remained several hours without a bandage.

16th May.—The swelling disappeared, and disease reduced to the condition of mild ophthalmia.

27th May.—The patient attended my clinical lectures without a bandage, and surprised every one who had seen him three weeks before in the state I have already described.

Early in June the ophthalmic symptoms re-appeared with much violence, a circumstance which I was at first inclined to attribute to the neglect of blisters and other secondary measures, but which a more rigid examination showed me to have arisen from the patient's own neglect of the local remedies prescribed in his treatment. These were therefore resumed with renewed perseverance, and in a few days the relapse was nearly terminated. After the symptoms had abated in violence, the patient commenced the employment of the ioduretted mineral water, and in this, associated with sulphureous baths, he continued for four months." 103.

### III. Scrofula of the Cutaneous Tissue.

Case 3. "Eloi Macaire, æt. 22, born of unknown parents, admitted April 19, 1830. Having been first placed in one of the surgical wards, he was transferred to me by my colleague Dr. Cloquet, who, regarding him as incurable by the ordinary modes of therapeutics, wished to afford me a new opportunity of testing the powers of iodine.

The patient was literally covered with old scars and scrofulous ulcers still in an open state. Both sides of the face and neck were invaded by wide, deep soft ulcers, with soft gelatinised edges, extending from the ears to the chin and the base of the sternum. The surface of this vast ulceration presented irregularities, in which were clearly perceptible the three principal sores of which it was composed. The base of these ulcers was tubercular, and surrounded with indurated cellular tissue, rendering the movements of the lower jaw difficult and painful. The head and cervical vertebræ in fact seemed but to form one piece. The separation of the jaws was so limited that a two-sous piece could not be introduced between the teeth. Mastication was consequently impossible, and the patient could only swallow liquid aliments, and even these with much difficulty and pain.

Behind the middle region of the right sterno-mastoid muscle, there was an oval ulcer two inches long, with a tubercular base. Above the humeral extremity of the right clavicle was a tumour larger than the closed hand, communicating a sense of fluctuation, passing under the clavicle and projecting on the chest. The unity of the abscess was proved readily by pressure above and below the clavicle.

Beneath this cyst a bag-shaped ulcer three inches long extended obliquely from the left towards the xyphoid cartilage. This was the primary ulcer which had existed since the age of three years, and had never been healed. The long duration and aspect of this sore, and especially its resistance to the numerous remedies tried for its relief, led to the belief that it was kept up by the caries of the ribs and sternum, and had induced several surgeons to propose the trephining of these bones.

To conclude this catalogue of ulcerations, the right axilla was occupied by a tubercular tumour presenting two ovoid ulcers placed one above the other, the inferior advancing rather to the front of the chest. These two ulcers had existed since the patient was nine years old. Those of the face and neck were but of four months' duration, and the indolent clavicular abscess had commenced but two months before the patient's admission into the Hôpital St. Louis. The symptoms underwent an annual exacerbation in June, and the suppuration was then much increased.

The abdominal parietes, the lower part of the left side of the chest, the neck and limbs, presented numerous scars of the worst character.

Macaire had passed almost his whole life in different hospitals and asylums. Having exhausted all the resources of the first establishment he was received into, he passed five months in the Civil Hospital of Lisle, without the slightest advantage. The organic lesions, already so numerous and so severe, and which had produced great debility and emaciation, were but too effectually assisted by the deep moral dejection of this unhappy young man, who since his birth had only experienced the most perfect state of disease, pain, misery, and destitution.

May 12, 1830. Ioduretted treatment. The sub-clavicular abscess was punctured, and there escaped a quantity of pus, or rather of softened tubercular matter, readily recognizable by its purulent and cheesy aspect. A solution of iodine was injected into the cyst and allowed to remain therein some minutes. The ulcers were dressed with charpie, strongly charged with the proto-ioduret of mercury. He was placed also on the use of the ioduretted mineral water, and he was ordered three sulphureous baths weekly.

26. Parietes of the abscess adherent; generally improved.

June 15. Ulcers of the face and neck cicatrized. The only remaining sore was that corresponding to the middle region of the sterno-mastoid muscle. The thoracic and axillary ulcers were replaced by well-conditioned scars. For three weeks he had recovered the free movements of his head and jaws, of which latter circumstance he availed himself with much pleasure.

July 10. Respiration embarrassed, cough, diminished appetite, sterno-mastoid ulcer suppurates pretty well. I had retarded the cure of this ulcer for some time, regarding it as a natural and beneficial issue. The analogy of these symptoms to those witnessed in the case of Jarry, decided me to use the same treatment, and it was attended with equal success. In this case also the touching of the ulcers, in the second fortnight of their treatment, with the concentrated solution of iodine, was attended with such striking local improvement, that it was perfectly visible from day to day, so that I was obliged to restrain it very soon, lest the sudden suppression of the ulcers should give rise to any dangerous effects.

25. Appetite good, he sleeps well, and feels happy. His only remaining ulcer was dressed once daily with the ointment of the proto-ioduret of mercury; his daily dose of iodine in solution was three quarters of a grain. The cicatrices were touched twice a week with the rubefacient solution, or caustic iodine, in order to diminish their redness, smoothness, and prominence.

October 25. In every respect as well as possible. The cicatrices were excellent, and no longer could convey any notion of the disease from which they resulted. Up to this time the local and internal treatment had been persisted in. The last ulcer was now healed, or very nearly so, its re-opening being encouraged from time to time for reasons already specified.

Dec. 31. Local treatment neglected for two months. The mineral water was continued in order to confirm the cure. No relapse has since taken place, and every thing seems to promise permanent good health." 111.

### IV. Scrofula of the Cellular Tissue.

Case 4. " Victor Auguste Dubois, joiner, aged 18, admitted into the Hôpital St. Louis, 24th August, 1830. His constitution appeared to be soft and weak; his skin very white, but scattered over with red spots; his hair was red, his chest narrow, and his respiration habitually impeded; his father and mother had not, as far as we could learn, been affected with scrofula, but of eight brothers and sisters four were manifestly tainted with that disease. Since infancy he had been afflicted with impetigo of the scalp and obstinate ophthalmia. Having come to Paris at the age of 16 he was attacked with chilblains, which rendered him lame of the right foot. At 18, after having been chilled, he experienced a dull pain under the right angle of the lower jaw, where an enormous abscess shortly formed, the progress of which was much accelerated after the application of ten leeches.

Eight days after this I saw the patient. The right cervical region was completely occupied by a knotty hard tumour. The superjacent skin was not altered in colour, there was no local heat. The tumour pressed most painfully on the subjacent parts, which it displaced considerably, although it projected externally to the volume of both hands. The head was thrown towards the left shoulder; the skin was extended and painful, though unyielding to the touch; the œsophagus and trachea were so compressed that respiration and deglutition were greatly embarrassed, and the patient could consequently swallow nothing more solid than bouilli.

*Treatment.* Frictions morning and evening, with the ointment of the proto-ioduret of mercury; ioduretted poultices after the frictions; mineral water for internal use; two or three sulphureous baths weekly.

9th September.—Tumour punctured where the skin seemed thinnest, &c. A quart of pus, mixed with albuminous flakes, was discharged. An ioduretted solution was injected into the empty cyst. Immediately after the puncture, the effects of the under-compression disappeared, and the patient swallowed and breathed without difficulty.

20th September.—Upper part of the opening cicatrized. Another puncture was required. The discharge having become crusty, the edges of the openings were thrice or four times touched with the caustic iodine, and the patient left the hospital cured on the 9th October, with no sequel of the disease but a slight hardness of the parietes of the cyst, which had contracted adhesion to the cellular tissue beneath." 117.

### V. Scrofula of the Bones.

Case 5.—" *White Swelling of the Right Knee—three Fistulous Ulcers—Hypertrophy and Induration of the Soft Parts of the Internal Half of the Thigh—Cure in Three Months.*

Louis Nicolas Irdot, æt. 26, carter, of small stature and sufficiently robust constitution, was admitted into the Hôpital St. Louis, Salle St. Jean, on the 24th March, 1830. His father, a street-porter, with the exception of tender eyes, and occasional ophthalmia, enjoyed good health; his mother also was free from habitual disease. Of fourteen children, eleven died in infancy; there remained a female, æt. 30, in good health, a brother, æt. 14, of weakly constitution, and the third, the subject of present notice. From his infancy he presented symptoms of scrofula; he had been harrassed with chilblains and indispositions of every kind, aggravated by deprivations and mendicity during his youth. At 15, he laboured at country work to the decided improvement of his health. At 22, after having lain on the ground while in a state of profuse perspiration, he experienced pains and swellings in both knees. This attack lasted four months. Two years after it returned without evident cause. It yielded readily in the left knee, but was aggravated proportionately in the right, which had been eighteen months affected when we saw him for the first time.

The right lower extremity was then disfigured by a hard fusiform tumour occupying the lower half of the thigh and the knee. At each side of the joint was a fistulous triangle. The soft parts in general were indurated, and approaching to the adipocirous state observed as a sequel to white swelling of the knee. The periosteum, and probably the femur, were enlarged, but the fistulous canals did not terminate on any carious surface. The leg was flexed on the thigh at a very obtuse angle, and its complete extension was impracticable. At the external surface of the thigh, close beneath the great trochanter, there were numerous cicatrices, which had long been fistulous, like those still existing at each side of the knee. The lymphatic ganglia of the right groin were slightly swelled, but there were no tubercles in any other part of the body.

When the iodine treatment was commenced the patient had been eighteen months bed-ridden. Sulphureous baths were also ordered, and he was obliged to take moderate exercise in the hospital walking-grounds. This method was attended with the most beneficial and rapid results. In six weeks he could walk with facility, and in three months he was cured.

The iodine practice was followed for six weeks after his cure, when he left the hospital in excellent health and spirits." 135.

The above are sufficient specimens of these cases, which have been selected rather for their brevity than for any other reason—certainly not on account of their exhibiting more the success of the remedy than the others which are passed over.

### EXERCISE IN WHITE SWELLING.

There is a chapter on the above subject, which the translator has thought it necessary to condense considerably. As this condensation occupies little more than a page of the work, we shall here quote it.

"It is unnecessary to call attention to that part of the treatment in the preceding cases, by which the ordinary doctrines and practice of medicine were contradicted—patients afflicted with white swelling of the ankle, knee, or hip-joints, being directed to walk in the ward or open air during the entire course of the iodine treatment.

I may venture, nevertheless, to solicit the notice of practitioners to the results of my general experience, in which I never observed any accident or inconvenience to result from this innovation ; of seventy-six scrofulous patients at present in my wards (30th April, 1831) there are thirty-two who, if treated according to the too general custom, would be restricted to absolute confinement to bed. Under my direction they walk daily in the hospital promenade, in the same manner as the different individuals afflicted with other forms of the malady.

The study of scrofula, as regards its causes and diagnosis, denotes that this disease has for its general character and original weakness which arrests the development of organs, but which renders them subsequently subject to a sudden and exaggerated increase. Rest has ever been regarded as a debilitating agent ; it is the ordinary associate of all antiphlogistic systems of treatment. The most vigorous and robust constitution would inevitably be weakened, and brought to a state of etiolation by long-continued repose. If rest thus debilitates the vigorous, still more should an indvalid, of primary weak constitution, be enfeebled by its operation, and his malady proportionately increased.*

But the matter is not one of argument alone; visit those patients confined to bed for six months, and on a debilitating regimen. They are pale, emaciated, weak, and depressed. I admit that the motion of a diseased joint is attended with some inconvenience, but the advantages derived from it are great beyond all proportion. In fine, for three years that I have followed this method, I have never been induced to change it, or even modify it, but for a transitory period in some unusual cases." 150.

We wish the ingenious translator had favoured us with his explanation of the utility of exercise in white swelling. For our own parts we are much disposed to agree with M. Lugol, and attribute the improvement to the air and exercise taken daily in the promenade of the hospital, instead of passing day and night in the tainted atmosphere of the ward, without the movement of a muscle to promote circulation or digestion—and without the advantage of any exhilaration from the association of convalescents in a public walk.

The third chapter of this division of the work, is on the mode of prescribing the preparations of iodine. In his first memoir he objected to the tincture and syrup for internal use, in consequence of the precipitation which he supposes to take place in the stomach. He therefore preferred the *perfect* solution in distilled water. But this *perfect* solution requires a large quantity of water, which is a great inconvenience in prescription. Another disadvantage is the decomposition of the solution when exposed to the light, and its deterioration thereby. For some time past, therefore, M. Lugol has dissolved the iodine in a solution of potash. We subjoin a tabular view of the solutions he is now in the habit of using, graduated in three different proportions, so that the iodine may be given internally in the progressive dose of half a grain, three-fourths of a grain, or four-fifths of one daily.

" IODURETTED MINERAL WATER.

| | No. 1. | No. 2. | No. 3. |
|---|---|---|---|
| ℞. Iodine............... | gr. $\frac{3}{4}$ | gr. i. | gr. i$\frac{1}{4}$ |
| Hydriodate of Potash | gr. 1$\frac{1}{2}$ | gr. ij | gr. ij$\frac{1}{2}$ |
| Distilled water..... | ℥viij | ℥viij | ℥viij." |

This solution is perfectly transparent, of a beautiful orange colour, and keeps for a considerable time. Children will take it readily when mixed with a little sugar, which should be added only at the time of exhibition. M. L. commences the treatment with half a grain of iodine—that is two-thirds of the mixture No. 1, per diem. In the second fortnight he gives the entire of this formula, and during the fourth fortnight, he gives a grain of iodine daily, till the conclusion of the trial.

" Another and advantageous form of preparing this mineral water on a larger scale is, by first making a concentrated solution of iodine in hydriodate of potash, and then diluting it with a sufficient proportion of water; thus,

---

* " M. Lugol will pardon me for remarking here, that I consider his facts much more conclusive than his arguments on the point in question. That exercise has effected much good in the cases before us I entertain no doubt; but that M. Lugol has satisfactorily accounted for the cause of the improvement I must be permitted to deny."—Translator's Note.

℞. Iodine . . . . . . . . . . . . . . . . . . . . . . . ℈j.

Hydriodate of potash . . . . . . . . . . . . . ℈ij.

Distilled water . . . . . . . . . . . . . . . . ℥vij.

This solution contains one twenty-fourth of iodine; poured into 16 pounds of distilled water, it forms thirty-two bottles of eight ounces of the mineral water No. 1. It is easy to understand that by diminishing the distilled water one-fourth we compose No. 2, and by using three-fifths of the quantity of water we obtain No. 3." 167.

The concentrated solution above-described serves for exhibition in drops once or twice a day—a mode of prescription very useful in private practice. We may commence with six drops twice a day, in a little water, gradually increasing the dose to 30 or 36 drops. Every week the dose should be augmented by two or three drops. The above is for the adult, of course.

In the formula for ioduretted ointment he has made little change—only a trifling reduction in the quantity of iodine in No. 1, in order to render it more suitable to tender skins. The prescription now stands thus:—iodine, 12 grains—hydriodate of potash, ℈iv.—hog's lard, ℥ij., *misce*. These ointments do not keep well. The following is the form which M. Lugol gives for a combination of iodine with mercury, under the term—

OINTMENT OF PROTO-IODURET OF MERCURY.

| | | | |
|---|---|---|---|
| ℞. Proto-ioduret of mercury . . . . | ℈ij. .. | ℈iij. .. | ℈iv. |
| Fresh lard . . . . . . . . . . . . . . . . | ℥ij. .. | ℥ij. .. | ℥ij.* |

This ointment is of a canary yellow colour, and the author was first led to its use by the syphilitic aspect of the esthiomenic form of scrofula, where its good effects were so conspicuous that he extended its use to all cases of external scrofulous disease. This ointment causes little or no pain in ordinary cases.

RUBEFACIENT SOLUTION OF IODINE.

℞. Iodine . . . . . . . . . . . . . . . . . . . . . . . . . . . . ʒiv.
Hydriodate of potash . . . . . . . . . . . . . . . . ℥i.
Distilled water . . . . . . . . . . . . . . . . . . . . . ℥vj.

This solution should be kept in a glass-stopped bottle, as it rapidly corrodes cork. It is very useful in cases where scrofulous surfaces require stronger excitement than usual—for example, the eye-lids and angles of the eye in obstinate chronic ophthalmia, coryza, and other forms of scrofulous disease in the nasal fossæ. It is most conveniently applied by means of pledgets of fine charpie. M. L. avers that its application to cicatrices has rendered them smoother and less livid. Local baths for the hands, arms, chin, &c. may be easily prepared, by adding the above solution to a sufficient quantity of warm water, taking care to employ wooden boxes for that purpose. Cataplasms may also be made with this solution and linseed-meal. The author uses this form in cases of hard tubercles, which obstinately resist other applications.

* The composition of the proto-ioduret of mercury will be given a little farther on.

### Caustic Iodine.

The most concentrated solution of iodine which can be made, is composed of one part of water, one part of the hydriodate of potash, and one part and a half of iodine. This solution induces small scars on the parts touched, similar to that produced by the nitrate of silver. In many cases it is more successful than friction—and in all, it may be advantageously associated with frictions. He touches excessive granulations on the eye-lids and nasal fossæ with the solution—and also the red hypertrophied skin surrounding certain scrofulous ulcers and tubercles.

"The celerity, in short, with which it improves the appearance of the soft and fungous tissues in these cases almost surpasses imagination: sometimes indeed, the ulcers are healed too soon; that is, closing before a sufficient change is worked on the general constitution. In the esthiomenic scrofula the pustules are touched with the caustic iodine, the rubefacient solution having been used a short time previously. The application may be made twice or thrice a week, sometimes even daily, when the surfaces are extensive, and can only be touched in minute proportions at a time." 176.

We must now notice the Appendix by the Translator. In this, he has made a selection from the French periodicals, of the cases published within the last six months—some of them valuable only as corroborating M. Lugol's statements—others, from the novel and apparently efficacious combinations of the remedy with other powerful therapeutic agents. The chemical history of the compounds of iodine has lately received some curious and important additions, in the labours of M. Caventon and others. These are taken advantage of by the translator. In the first section of this appendix the translator has brought forward a succinct analysis of cases treated with simple iodine, or hydriodate of potash and opium—some with ioduret of mercury—and others with the ioduret of lead. In the second section, he has described fully the chemical properties of iodine, hydriodate of potash, and the metallic iodurets just mentioned, including an account of the adulterations to which they are commonly subjected. The cases we are unable to notice. It appears from them that the combination of opium with iodine has often been highly beneficial—a fact which we can readily believe, knowing, as we do, how much the soothing influence of opium assists the virtues of many other medicines, especially mercury.

### Therapeutic Effects of the Ioduret of Lead.

"The ioduret of lead appears to be by far the most interesting of any of the metallic compounds of iodine yet tried in practical medicine. Its introduction is but of very recent date, and is due to the researches of two Parisian physicians of great promise, MM. Cottereau and Verdet de Lisle. Having witnessed the singular rapidity with which absorption took place in a tumour to which a soap accidentally containing the ioduret of lead was applied, they immediately commenced an extensive investigation into the therapeutic properties of the compound, and have obtained results, certainly, of no trivial importance. The following passage, quoted from a letter published in one of the French periodicals, by MM. Cottereau and De Lisle, leads us to expect much from the repetition of clinical experiments on this subject:—

'We thank you for calling the attention of your readers to this new medicine; for we have ascertained, in an incontrovertible manner, that of all the preparations of iodine this is the most efficacious, and promises the most prompt and constant action. It is, moreover, free from the inconvenience of creating the cutaneous inflammation which the simple iodine and hydriodates occasion. The proofs of this we shall afford by publishing the cases we have collected, as well in Paris as in the hospitals and departments,—cases, the majority of which had previously been ineffectually submitted to the action of iodine in other forms. Many other practitioners, who have prescribed the remedy at our desire, participate fully in these opinions.'—*Gazette des Hôpitaux, 30th June,* 1831.

Extensive trials of this substance have been made at the Hôpital des Enfans by M. Guersent, and at La Pitié by M. Velpeau. M. Guersent has not yet published any account of his researches; but Dr. Haas states, in the *Journal Hebdomadaire,* No. 31, that 'he has already witnessed the complete and rapid success of the ioduret of lead, in several cases treated by M. Guersent at the Hôpital des Enfans.' I subjoin an outline of some cases treated by M. Velpeau, at La Pitié, and mentioned by him in his clinical lectures." 206.

The chemical properties of iodine and hydriodate of potash being sufficiently explained in the Dublin Pharmacopœia, where they are officinal, and in various elementary works, the translator dwells chiefly on the means by which their purity may be ascertained, and for his remarks on this important subject, we must refer to the work itself. Happily, iodine is now reduced in price to one shilling and sixpence, or two shillings per ounce, which will tend to check the temptation to adulterate it. But the hydriodate of potash, the translator avers, is so variously and ingeniously sophisticated, that the practitioner who is not aware of their existence and the mode of detection, need never attempt to place a patient under Lugol's treatment, with any reasonable prospect of success! This is a melancholy reflection, but we fear it is founded in too much truth. It appears that chemists and druggists are in the constant habit of mixing large proportions of muriate of soda, carbonate of potash, &c. with hydriodate of potash. One specimen which Dr. O. S. examined contained 64 per cent. of the carbonates alone. We must endeavour to make room for the following extract.

HYDRIODATE OF POTASH.

| PURE. | IMPURE. |
|---|---|
| 1. Dissolves iodine in the cold, forming an active ioduretted hydriodate of potash for internal use. | 1. Does not dissolve iodine in the cold. |
| 2. When warmed with iodine and diluted with water forms an *active* bath, which excites powerful local action. | 2. When warmed with free iodine converts it into the hydriodate of potash, a compound proved by Lugol to be nearly inert as a local or general bath. |
| 3 Affords by double decomposition pure iodurets of lead or mercury for internal or external use. | 3. Affords by double decomposition carbonates, chlorides, and sulphates of lead or mercury, compounds either inert or opposed in their action to the iodurets of these metals. |
| *Conclusion.* When pure, a valuable medicinal and pharmaceutical agent. | *Conclusion.* Impure, possessed of no medicinal or pharmaceutical value whatever. |

### Ioduret of Lead.

"This compound is prepared by adding a solution of 100 parts of the hydriodate of potash to a solution of 75 parts of the acetate of lead. In the preceding section I have given a diagram illustrative of the mutual decomposition which takes place.

The ioduret of lead is a fine yellow powder, partially soluble in acetic acid and in alcohol; insoluble in cold, but perfectly soluble in hot water, from which it crystallizes in fine hexagonal plates. One hundred parts of this compound consists of 54.9. iodine, 45.1. lead. The discovery of the solubility of the ioduret of lead was first made by M. Polydore Boullay in 1827. It then, however, attracted but little attention; and was completely forgotten when M. Caventon again casually noticed the circumstance, and placed it prominently before the public.

The ioduret of lead has very recently been subjected to an elaborate analysis by M. Henry (fils), '*Journal de Pharmacie,* Mai 1831,' and 100 parts found to consist of iodine 54.9. lead 45.1.

When the ioduret of lead precipitated in the cold is acted on by boiling water, it is dissolved with the exception of a minute whitish grey residuum, which appears to consist of an ioduret of lead with excess of base. Various experiments also show that the composition of the pulverulent ioduret is by no means constant in its nature, occasionally containing free iodine and occasionally an excess of lead. The crystallized product deposited from warm water, should therefore alone be employed for pharmaceutical or medicinal purposes." 215.

### Iodurets of Mercury.

Iodine, observes the translator, forms two compounds with metallic mercury—the first of a fine canary yellow—the second of a beautiful carmine colour. The first of these compounds is prepared by adding a solution of the hydriodate of potash to a solution of *proto* salt of mercury. The second is made by adding the hydriodate to a *per* salt of the same metal.

"In the preparation of the proto-ioduret by double decomposition, great care is necessary that the salt of mercury should contain no per-oxide, otherwise a deuto-ioduret would be formed, a compound analogous in its properties to the bichloride or corrosive sublimate. By observing the following directions, however, the proto-ioduret will be obtained perfectly pure.

Dissolve, without applying heat, a sufficient quantity of pure mercury in one part of nitric acid diluted with three parts of distilled water, and add mercury until no more be dissolved. A proto-nitrate of mercury is thus formed which frequently shoots into a mass of white crystals. Any excess of metallic mercury is to be separated by inclining the vessel and allowing it to run off, the solution containing the crystals is then to be diluted with distilled water until they are perfectly dissolved; a pure proto-nitrate of mercury is thus obtained, the formation of the per-nitrate being only occasioned by the application of heat and the use of too concentrated nitric acid.

Hydriodate of potash is to be added to this solution as long as any precipitate occurs. Filtration is then to be performed, the matter remaining on the filter to be well washed with distilled water, and dried in a water bath. As thus prepared, the proto-ioduret of mercury is a fine yellow powder, quite insoluble in water at any temperature." 217.

After having given so ample an analysis of this volume, it is hardly necessary to say that the subject is so exceedingly important that no medical man should fail to place the work in his library for constant reference and study.

It is decidedly the most valuable monograph which has issued from the press for many years—being entirely practical, and founded on accurate, as well as authentic clinical observations and experiments. The numerous quotations which we have made, in the course of this article, will enable every English reader to judge of the manner and construction of the English version. The translation is singularly faithful, though not always strictly literal; and, with very few exceptions, it reads as fluently and as gracefully, as a studied original composition. The style befits a scientific subject—clear, perspicuous, unaffected, yet energetic. We need say no more.

